# Integrative Analysis Identifies a TNFα-Derived Gene Signature for Predicting Prognosis, Tumor Immunity, and Treatment Sensitivity in Gastric Cancer

**DOI:** 10.3389/fgene.2022.882519

**Published:** 2022-06-02

**Authors:** Ke Wang, Lina Qi, Hua Sun, Min Diao, Lin Yang

**Affiliations:** ^1^ Nursing Department, The Second Affiliated Hospital of Shandong First Medical University, Tai’an, China; ^2^ Philippine Women’s University, Manila, Philippines; ^3^ PICC Clinic, Taian City Central Hospital, Tai’an, China; ^4^ Hand and Foot Surgery, The Second Affiliated Hospital of Shandong First Medical University, Tai’an, China; ^5^ Pediatric Intensive Care Unit, The Second Affiliated Hospital of Shandong First Medical University, Tai’an, China

**Keywords:** gastric cancer, TNFα, model, prognosis, tumor immune microenvironment, treatment sensitivity

## Abstract

**Objective:** TNF-α is an essential pro-inflammatory cytokine in the tumor microenvironment of gastric cancer (GC), possessing a key biological and clinical impact. Here, we conducted an integrative analysis of the role of TNFα-derived genes in GC prognosis and precision medicine.

**Methods:** We pooled transcriptome and clinical features of GC patients from TCGA and GSE15459 projects. TNFα signaling was quantified through the ssGSEA algorithm, and TNFα-derived genes were screened with WGCNA. Thereafter, a LASSO model was established. The somatic mutation was analyzed across GC specimens. Immune cell infiltrations were inferred through ESTIMATE and ssGSEA algorithms, followed by measuring the immune checkpoint expression. AKR1B1, CPVL, and CTSL expressions were measured in gastric mucosal cells GES-1 and GC cells (HGC-27, MKN-28, and AGS) through RT-qPCR and Western blotting.

**Results:** A TNFα-derived gene signature (containing AKR1B1, CPVL, and CTSL) was developed for GC. A high-risk score indicated more undesirable OS, DFS, DSS, and PFS outcomes. Time-independent ROC curves and multivariate cox regression models confirmed that the signature reliably and independently predicted GC prognosis. Additionally, risk scores displayed significant correlations to more severe histological grades and pathological stages. A low-risk score was characterized by increased somatic mutation, while a high-risk score was characterized by immune and stromal activation, enhanced immune cell infiltrations, and increased expression of immune checkpoint molecules. Experimental results confirmed the significant upregulation of AKR1B1, CPVL, and CTSL in GC cells.

**Conclusion:** Collectively, stratification based on the TNFα-derived gene signature might enable GC patients to predict prognosis, benefit from immunotherapy, and assist in formulating novel therapeutic regimens.

## Introduction

Gastric cancer (GC) ranks the fifth most frequently diagnosed cancer and is the third major cause of cancer deaths across the globe (Kim et al., 2021; Saeed et al., 2021; Zhang et al., 2021). In accordance with the latest global cancer statistics, there were over one million newly diagnosed cases and approximately 7,83,000 death cases of GC in 2018 (Bray et al., 2018). Despite the declined morbidity and mortality within the past years, GC remains a severe global health issue. Treatment regimens have been challenged due to the complexity and controversy of GC progression (Wang et al., 2021). Currently, surgical, chemo-, radio- and targeted therapies have become the major therapeutic approaches (Yu et al., 2021). The AJCC staging system and histological classifications represent the most important tools for stratifying, classifying, and treating GC patients (Wu et al., 2021). Extensive heterogeneity has been found in GC, indicating that it is of importance for novel stratifications and identification of other important factors to stratify patients more precisely for better guiding clinical therapy and improving clinical outcomes (Qiu et al., 2020).

Growing pieces of evidence demonstrate the important implications of tumor necrosis factor-alpha (TNF-α) in gastric carcinogenesis, which is an essential proinflammatory cytokine in the tumor microenvironment of GC and the main cytokine of cancer pain (Ishimoto et al., 2017; Baj et al., 2020; Zhuang et al., 2020). For instance, GC cell-derived TNF-α triggers the IL-33 expression in cancer-associated fibroblasts through the TNFR2-NF-κB-IRF-1 axis (Chen et al., 2020; Zhou et al., 2020). The TNF-α and NF-κB signaling pathways are mutually positive feedback regulations. TNF-α activates the NF-κB pathway, which is significantly related to cancer pain. Meanwhile, this activated signaling pathway can promote the transcription and synthesis of TNF-α, which in turn leads to more serious cancer pain (Yang et al., 2020). Tumor-associated macrophages induce the PD-L1 expression in GC cells partly *via* TNF-ɑ signaling (Ju et al., 2020). Elevated intratumoral mast cell fosters immunosuppression and GC progression *via* the TNF-α-PD-L1 pathway (Lv et al., 2019). Hence, it is of great significance to uncover the biological and clinical impact of TNF-α-derived signatures in GC. Based on mRNA expression profiles derived from TCGA, this study developed a TNFα-derived gene signature for predicting the prognosis and immunotherapeutic responses, as well as assisting in formulating novel therapeutic regimens.

## Materials and Methods

### Patient Cohort and Data Acquisition

RNA sequencing data (in fragments per kilobase per million (FPKM)) of TCGA-STAD (stomach adenocarcinoma) cohort were curated from the Genomic Data Commons (GDC) data portal (https://portal.gdc.cancer.gov/). Thereafter, the FPKM format was converted to the transcripts per kilobase million (TPM) format for further analysis. Clinical features of GC patients were also harvested from TCGA project. The GSE15459 dataset was downloaded from the Gene Expression Omnibus (GEO) repository (https://www.ncbi.nlm.nih.gov/gds/), which was used as the external validation set.

### Collection of Gene Sets of Tumor Necrosis Factor-α Signaling

The gene sets of TNFα-signaling were curated from the Molecular Signatures Database (MSigDB; http://www.broadinstitute.org/msigdb) (Liberzon et al., 2015). Single-sample gene set enrichment analyses (ssGSEA) derived from the gene set variation analysis (GSVA) package were presented for quantifying the activities of TNFα signaling across GC specimens (Hänzelmann et al., 2013). The ssGSEA ranked the mRNA expression in each specimen and used empirical cumulative distribution function of genes in the signature and the remaining genes to produce an enrichment score. The ssGSEA score was normalized through the Z-score.

### Weighted Gene Co-Expression Network Analysis

The WGCNA package was adopted for performing co-expression analysis (Langfelder and Horvath, 2008). The expression profiling of the first 5,000 genes according to SD was included for the WGCNA. The soft-thresholding power ß was set as 3 with the pickSoftThreshold function. Additionally, the scale-free *R* ^*2*^ = 0.85 calculated with the softConnectivity function was set as the soft-thresholding parameter for ensuring a scale-free topology network and producing a TOM matrix. Thereafter, co-expression modules were clustered. Pearson correlation analysis was carried out between the co-expression modules and TNFα score. Moreover, the correlations between module membership and gene significance were plotted. Genes in the co-expression module that presented the strongest correlation strength with the TNFα score were deemed as TNFα-derived genes.

### Establishment of the Tumor Necrosis Factor-α–Derived Genomic Model

Univariate cox regression analysis was conducted for screening prognostic TNFα-derived gene signatures (*p* < 0.05). Thereafter, this study input the aforementioned genes into the Least Absolute Shrinkage and Selection Operator (LASSO) analyses with the glmnet package (Engebretsen and Bohlin, 2019). Characteristic genes were screened in accordance with the optimal *λ* value. The TNFα-derived risk score was determined following the formula: risk score = ∑ X i * coef i, in which coef i was the coefficient, and X i was the mRNA expression of each characteristic gene. GC patients were randomly separated into training and testing sets with a 1:1 ratio. With the median value of the risk score, patients were divided into high- and low-risk groups in each dataset. Kaplan–Meier curves of overall survival (OS), disease-free survival (DFS), disease-specific survival (DSS), and progression-free survival (PFS) were conducted between high- and low-risk groups. Survival differences were estimated with log-rank tests. Time-independent receiver operating characteristic (ROC) curves were presented for evaluation of the efficacy of the risk score in predicting GC OS outcomes. Uni- and multivariate cox regression models were constructed for screening independent prognostic factors of GC.

### Development of a Nomogram

The TNFα-derived gene signature and clinicopathological characteristics (age, T stage, N stage, M stage, and pathological stage) were input into the nomogram model in TCGA-STAD dataset. Calibration curves, ROC curves at 5-, 6- and 7-year survival, and decision curve analyses (DCA) were presented for evaluating whether this nomogram was useful as an ideal model.

### Functional Enrichment Analyses

GSEA was presented for comparing activated hallmark gene sets between high- and low-risk groups in TCGA-STAD cohort (Subramanian et al., 2005). For each analysis, 1,000 gene set permutations were carried out. The hallmark gene sets curated from the MSigDB project were utilized as the reference set. Additionally, the ssGSEA score of hallmark gene sets was calculated across GC tissues.

### Estimation of TME-Infiltrating Immune Cells

Estimation of STromal and Immune cells in MAlignant Tumor tissues using Expression data (ESTIMATE) algorithm (Yoshihara et al., 2013) was utilized for evaluations of immune and stromal scores in accordance with mRNA expression signatures. Immune and stromal scores represented the tumor immune and stromal infiltrations within a bulk tumor. Thereafter, the ESTIMATE score was defined by combining immune and stromal scores within tumor tissues. Through ssGSEA, the abundance of immune cells was scored within tumor tissues in accordance with mRNA expression profiles.

### Prediction of Immunotherapy and Chemotherapy Responses

The T-cell dysfunction and exclusion (TIDE) algorithm (http://tide.dfci.harvard.edu/) was calculated for predicting the clinical responses to immune checkpoint inhibitors (Jiang et al., 2018). The immunophenoscore (IPS) was determined for the prediction of the responses to CTLA-4 or PD-1 inhibitors in accordance with the marker genes of MHC-relevant signatures, checkpoint molecules, immunomodulators, effector cells, and suppressor cells (Charoentong et al., 2017). All steps within the cancer immunity cycle that reflected the anticancer immune response were quantified through the ssGSEA algorithm (Chen and Mellman, 2013). The half-maximal inhibitory concentration (IC50) values of chemotherapeutic agents from the Cancer Genome Project (Geeleher et al., 2014b) were estimated utilizing the pRRophetic package (Geeleher et al., 2014a).

### Somatic Mutation Analyses

Somatic mutation profiling (mutation annotation format (MAF) files) of TCGA-STAD was analyzed with MuTect2 and visualized with the maftools package (Mayakonda et al., 2018). The tumor mutational burden (TMB) was determined through non-synonymous somatic mutations utilizing 38 Mb as the estimate of the exome size (Chalmers et al., 2017).

### Cell Culture

Human gastric mucosal cells GES-1 and human GC cells HGC-27, MKN-28, and AGS were purchased from the Chinese Academy of Sciences. All cells were maintained in Dulbecco’s modified Eagle’s medium (DMEM; Hyclone, United States) containing 10% fetal bovine serum (Gibco, United States), 100 U/ml penicillin sodium, and 100 μg/ml streptomycin (Hyclone, United States ). The cell culture flask was placed in a 5% CO_2_ incubator at 37°C.

### Western Blotting

Cells were washed twice lasting 2 min through PBS and resuspended by using radioimmunoprecipitation assay buffer at 4°C. The protein content was evaluated utilizing a BCA kit (Beyotime, China), in accordance with the manufacturer’s protocols. Then, 200 µl protein lysates were separated *via* 10% SDS-PAGE and transferred onto the polyvinylidene difluoride (PVDF) membrane. Thereafter, the membrane was incubated with TBST (TBS with 1% Tween 20) containing 5% BSA lasting 1 h at room temperature and subsequently incubated with primary antibodies targeting AKR1B1 (1/1000; ab192865; Abcam, United States), CPVL (1/1000; ab180147; Abcam, United States), CTSL (1/1000; ab200738; Abcam, United States), and GAPDH (1/10000; ab8245; Abcam, United States) overnight at 4°C. The membrane was washed by TBST lasting 5 min at room temperature, followed by incubation with horseradish peroxidase-conjugated goat anti-rabbit secondary antibodies (1/2000; ab7090; Abcam, United States) at 37°C lasting 1 h. Through an electrochemiluminescence detection kit (Bio-Rad, United States), the protein bands were developed, and the protein expression was tested with an X-ray film. The bands were quantified by ImageJ software.

### Reverse Transcription and Quantitative Real-Time PCR

Total RNA was extracted from cells utilizing RNeasy kits (Beyotime, China) and reverse transcribed with one-step RT-PCR kits (Beyotime, China) at 37°C lasting 30 min, in accordance with the manufacturer’s protocols. qPCR was conducted utilizing SYBR Green RT-PCR kits (Takara, China). The thermocycling conditions were as follows: 95°C lasting 5 min; 40 cycles of 95°C lasting 40 s, 60°C lasting 30 s, and 72°C lasting 30 s. The following primers were used for PCR: AKR1B1: 5′-TTT​TCC​CAT​TGG​ATG​AGT​CGG-3′ (forward), 5′-CCT​GGA​GAT​GGT​TGA​AGT​TGG-3′ (reverse); CPVL: 5′-TGG​AAG​GTG​ATT​GTT​TCG​CTG-3′ (forward), 5′-GTC​TCC​CTT​AGG​TGG​CAT​GGA-3′ (reverse); CTSL: 5′- CTT​TTG​CCT​GGG​AAT​TGC​CTC -3′ (forward), 5′-CAT​CGC​CTT​CCA​CTT​GGT​C-3′ (reverse); and GAPDH: 5′-GGA​GCG​AGA​TCC​CTC​CAA​AAT-3′ (forward), 5′-GGC​TGT​TGT​CAT​ACT​TCT​CAT​GG-3′ (reverse). The fold change in mRNA expressions was determined with the 2^−ΔΔCq^ method.

### Statistical Analyses

All analyses were executed through R (version 4.0.1) and GraphPad Prism (version 8.0.1) software. With Student’s or Wilcoxon test, comparisons between groups were conducted. Pearson’s or Spearman’s correlation test was utilized to evaluate the associations between variables. *p* < 0.05 was indicative of statistical significance.

## Results

### Quantification of the Tumor Necrosis Factor-α Score as a Prognostic Indicator and Identification of Tumor Necrosis Factor-α–Derived Genes

**FIGURE 1:**
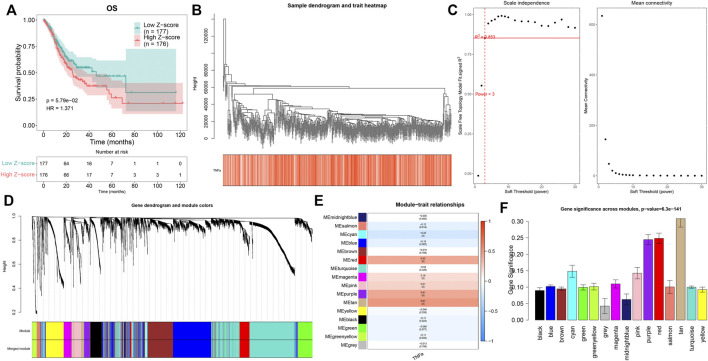
Quantification of the TNFα score as a prognostic indicator and identification of TNFα-derived genes. **(A)** Kaplan–Meier curves of OS for GC patients with high and low z-score of TNFα signaling. **(B)** Hierarchical clustering for detection of outlier samples. **(C)** Determination of scale independence and mean connectivity under diverse soft thresholds. The redline corresponded to 0.853. Soft-thresholding power was set as 3 after considering both scale independence and mean connectivity. **(D)** Hierarchical cluster analyses for detecting co-expression modules assigned by distinct colors. **(E)** Heatmap displaying the Pearson correlation of co-expression modules with the TNFα score. **(F)** Gene significance across co-expression modules.

Through the ssGSEA method derived from the GSVA package, we quantified the activities of TNFα signaling across GC tissues. In accordance with the median value of z-scores of TNFα signaling, we separated GC patients into high and low z-score groups. Kaplan–Meier curves demonstrated that GC patients with high z-scores displayed more undesirable OS outcomes than those with low z-scores (Figure 1A). This indicated that TNFα signaling might be linked to GC prognosis, which was consistent with previous research (Ju et al., 2020). This study employed the WGCNA approach to further identify TNFα signaling-derived genes in GC. First, the top 5,000 genes according to SD were included for co-expression analyses. The genes with similar expression patterns would be clustered into one module. Hierarchical clustering analyses indicated that there was no outlier among GC samples (Figure 1B). For constructing an appropriate scale-free topological overlap matrix, we calculated the scale independence and mean connectivity under diverse soft thresholds. Consequently, when soft thresholding was set as 3, the scale-free R^2^ was 0.853, indicating the constructed co-expression network met the scale-free topology criterion (Figure 1C). Thereafter, GC samples were clustered into 16 co-expression modules (Figure 1D). To determine the correlation between co-expression modules and the TNFα score as a phenotype, we carried out a Pearson correlation analysis. Our results uncovered that the “tan” module displayed the strongest correlation to the TNFα score (*R* = 0.51 and *p* < 0.0001; Figure 1E). Moreover, we compared the gene significance of each module with the TNFα score. In particular, we noted that the “tan” module presented the highest gene significance with the TNFα score (Figure 1F), indicating that the genes in the “tan” module were prominently associated with TNFα signaling. Herein, the 80 genes in the “tan” module were considered TNFα-derived genes, which are listed in Supplementary Table S1.

### Construction of a Tumor Necrosis Factor-α-Derived Gene Signature for Prediction of Gastric Cancer Prognosis

**FIGURE 2:**
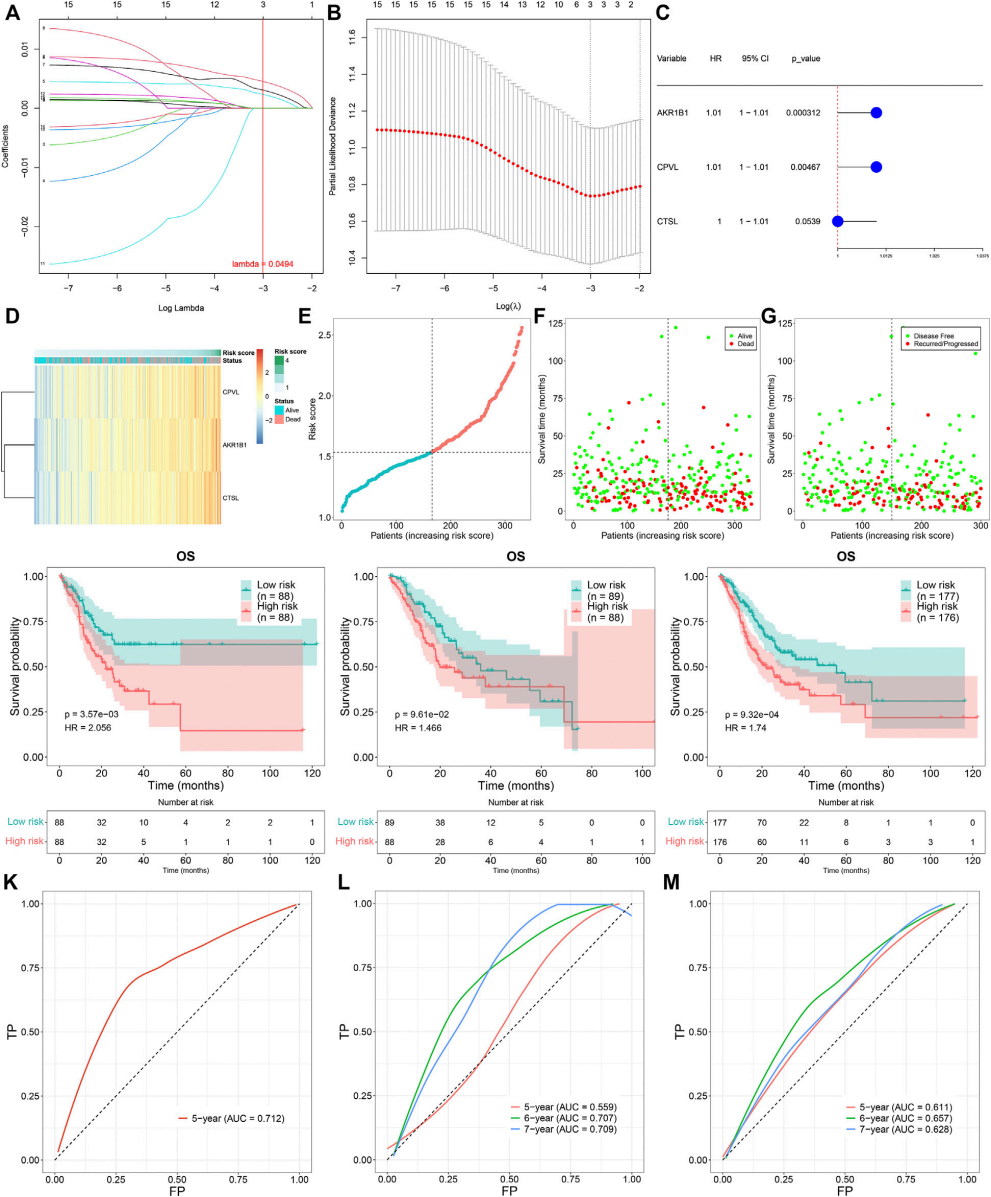
Construction of a TNFα-derived gene signature for prediction of GC prognosis. **(A)** LASSO coefficient profiling of prognostic TNFα-derived genes. The redline indicated the value determined by three-fold cross-verification. **(B)** Tuning parameter selection for the LASSO model. The partial likelihood of deviance was depicted against log (λ), in which *λ* was the tuning parameter. A partial likelihood deviance value was displayed, and error bars represented SE. A dotted vertical line was drawn at the optimal value through minimum and 1-SE criteria. **(C)** Univariate cox regression analyses of the associations of GC prognosis and characteristic TNFα-derived genes. **(D)** Heatmap visualizing the expressions of characteristic TNFα-derived genes in high- and low-risk groups. **(E)** Distribution of the TNFα-derived risk score across GC patients. The vertical dotted line represented the grouping cutoff. **(F)** Distribution of the survival status (alive and dead) among GC patients. **(G)** Distribution of the recurred and progressed status among GC patients. **(H–J)** Kaplan–Meier curves of OS outcomes for high- and low-risk GC patients in the **(H)** training set, **(I)** testing set, and **(J)** entire set. **(K–M)** ROC curves at 5-year, 6-year, and 7-year survival based on TNFα-derived risk scores in **(K)** training, **(L)** testing, and **(M)** entire sets.

To determine prognosis-related TNFα-derived genes, we conducted univariate cox regression analyses. Our results showed that 15 TNFα-derived genes displayed significant associations with GC prognosis (*p* < 0.05; Table 1). The aforementioned genes were input into LASSO analyses. With the optimal λ (0.0494), three genes (AKR1B1, CPVL, and CTSL) were retained following LASSO regularization (Figures 2A,B). Figure 2C showed the prognostic significance of AKR1B1, CPVL, and CTSL in GC. The risk score of each GC specimen was quantified utilizing the established formula: risk score = 0.00453439137355748 * AKR1B1 expression +0.0023541802071365 * CPVL expression +0.00307599022458496 * CTSL expression. With the increase in the risk score, the expressions of AKR1B1, CPVL, and CTSL were gradually increased in all GC patients (Figure 2D). In accordance with the median value of the risk score, GC patients were separated into high- and low-risk groups (Figure 2E). We noted there were more patients with the dead and recurred or progressed status in the high-risk group (Figures 2F,G). Thereafter, GC patients were randomly separated into two parts (1:1) for training and testing sets. Table 2 summarized the clinical characteristics of GC patients from training, testing, and entire sets. Our data demonstrated that high-risk patients presented more undesirable OS than low-risk patients in training (Figure 2H), testing (Figure 2I), and entire sets (Figure 2J). ROCs at 5-, 6- and 7-year survival confirmed that the TNFα-derived risk score was accurately and sensitively predictive of GC prognosis in training (Figure 2K), testing (Figure 2L), and entire sets (Figure 2M).

**TABLE 1:** Univariate Cox regression models identify prognostic TNFα-derived genes.

Gene	*p*-value	HR	AUC (3-year)	AUC (4-year)	AUC (5-year)	AUC (6-year)	AUC (7-year)
GPNMB	7.85E-05	1 + 1.81E-03	0.53	0.57	0.62	0.60	0.46
AKR1B1	2.14E-04	1 + 8.04E-03	0.58	0.60	0.56	0.57	0.57
CPVL	1.97E-03	1 + 5.41E-03	0.53	0.54	0.52	0.52	0.44
NPC2	3.39E-03	1 + 3.88E-03	0.54	0.56	0.50	0.61	0.55
CSF1R	3.81E-03	1 + 4.24E-03	0.52	0.56	0.58	0.59	0.45
MS4A6A	5.62E-03	1 + 7.67E-03	0.52	0.55	0.59	0.59	0.51
LHFPL2	1.39E-02	1 + 5.98E-03	0.53	0.55	0.60	0.53	0.46
CD163	1.53E-02	1 + 4.49E-03	0.51	0.54	0.58	0.58	0.41
SRGN	1.79E-02	1 + 8.23E-04	0.52	0.53	0.54	0.57	0.49
FCGR2A	1.86E-02	1 + 4.87E-03	0.55	0.57	0.64	0.61	0.47
ADAM28	2.05E-02	1 + 2.74E-03	0.49	0.52	0.51	0.55	0.49
CTSL	2.27E-02	1 + 3.79E-03	0.55	0.54	0.58	0.46	0.46
STAB1	3.29E-02	1 + 3.29E-03	0.55	0.57	0.57	0.56	0.57
CD14	3.56E-02	1 + 2.91E-03	0.52	0.53	0.54	0.58	0.43
TPP1	4.07E-02	1 + 1.47E-03	0.56	0.53	0.54	0.51	0.44

**TABLE 2:** Clinical characteristics of GC patients from training, testing, and entire sets.

Variable	Entire set (*n* = 353)	Training set (*n* = 176)	Testing set (*n* = 177)
Age	65.51 ± 10.62	65.17 ± 10.18	65.85 ± 11.07
Status			
Alive	207 (58.64)	106 (60.23)	101 (57.06)
Dead	146 (41.36)	70 (39.77)	76 (42.94)
Sex			
Male	228 (64.59)	112 (63.64)	116 (65.54)
Female	125 (35.41)	64 (36.36)	61 (34.46)
T stage			
T1	18 (5.1)	8 (4.55)	10 (5.65)
T2	74 (20.96)	37 (21.02)	37 (20.9)
T3	163 (46.18)	86 (48.86)	77 (43.5)
T4	94 (26.63)	43 (24.43)	51 (28.81)
Unknown	4 (1.13)	2 (1.14)	2 (1.13)
N stage			
N0	103 (29.18)	55 (31.25)	48 (27.12)
N1	96 (27.2)	51 (28.98)	45 (25.42)
N2	73 (20.68)	32 (18.18)	41 (23.16)
N3	71 (20.11)	30 (17.05)	41 (23.16)
Unknown	10 (2.83)	8 (4.55)	2 (1.13)
M stage			
M0	314 (88.95)	154 (87.5)	160 (90.4)
M1	23 (6.52)	13 (7.39)	10 (5.65)
Unknown	16 (4.53)	9 (5.11)	7 (3.95)
Pathological stage			
Stage I	48 (13.6)	23 (13.07)	25 (14.12)
Stage II	109 (30.88)	62 (35.23)	47 (26.55)
Stage III	146 (41.36)	65 (36.93)	81 (45.76)
Stage IV	35 (9.92)	17 (9.66)	18 (10.17)
Unknown	15 (4.25)	9 (5.11)	6 (3.39)

### Clinical Implication and External Validation of the Tumor Necrosis Factor-α–Derived Gene Signature in Gastric Cancer

**FIGURE 3:**
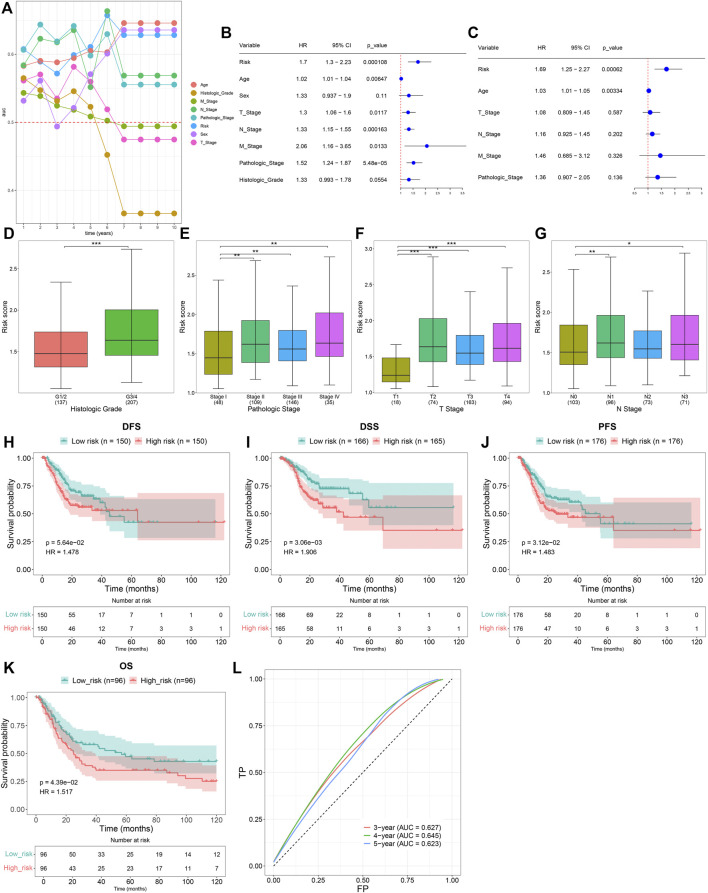
Clinical implication of the TNFα-derived gene signature in GC. **(A)** Time-independent ROC curves of the TNFα-derived risk score and conventional clinicopathological characteristics. **(B)** Univariate Cox regression analyses for the associations of the TNFα-derived risk score and conventional clinicopathological characteristics with GC prognosis. **(C)** Multivariate Cox regression analyses for evaluations of the predictive independency of the aforementioned factors in GC prognosis. **(D–G)** Distribution of the TNFα-derived risk score in distinct clinicopathological characteristics, containing the **(D)** histological grade, **(E)** pathological stage, **(F)** T stage, and **(G)** N stage. *p < 0.05; **p < 0.01; ***p < 0.001. **(H–J)** Kaplan–Meier curves of **(H)** DFS, **(I)** DSS, and **(J)** PFS for high- and low-risk GC patients. **(K)** Kaplan–Meier curves of OS for high- and low-risk GC patients in the GSE15459 dataset. **(L)** ROCs at 3-, 4-, and 5-year survival in the GSE15459 dataset.

Time-independent ROCs revealed that the TNFα-derived risk score displayed a prominent advantage in predicting GC prognosis (Figure 3A). Univariate cox regression analyses showed that the TNFα-derived risk score was indicative of an undesirable prognosis of GC (Figure 3B). Furthermore, multivariate cox regression analyses uncovered that the TNFα-derived risk score acted as an independent risk factor of GC outcomes (Figure 3C). Compared with G1/2, a higher risk score was detected in G3/4 patients (Figure 3D). Additionally, we noted that compared with stage I, there was a prominently increased risk score in stages II, III, and IV (Figure 3E). In comparison to the T1 stage, a significantly higher risk score was investigated in T2, T3, and T4 stages (Figure 3F). Also, N1 and N3 patients presented an increased risk score compared to those with N0 (Figure 3G). The aforementioned findings demonstrated that the TNFα-derived risk score was in relation to GC progression. Further survival analyses suggested that high-risk patients indicated poorer DFS (Figure 3H), DSS (Figure 3I), and PFS (Figure 3J) than low-risk patients. The clinical applicability of this model was further validated in the GSE15459 dataset. Consistently, the high-risk score predicted poorer OS than the low-risk score (Figure 3K). Additionally, ROCs at 3-, 4- and 5-year survival demonstrated that this model enabled the prediction of GC prognosis accurately and sensitively (Figure 3L).

### Establishing a Nomogram of Gastric Cancer Patients

**FIGURE 4:**
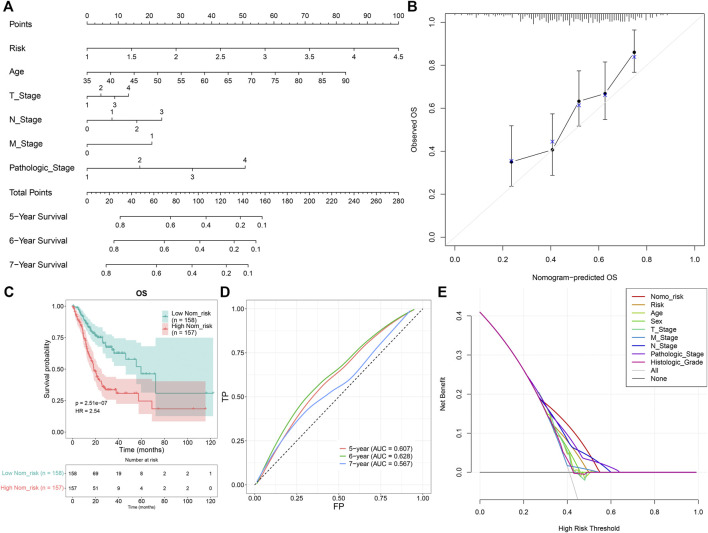
Development of a prognostic nomogram for GC patients. **(A)** Nomogram model integrating the TNFα-derived gene signature, age, T stage, N stage, M stage, and pathological stage for prediction of GC patients’ 5-, 6-, and 7-year OS probabilities. **(B)** Calibration curves for this nomogram-predicted and observed OS outcomes. The 45-degree line meant the ideal prediction. **(C)** Kaplan–Meier curves of OS for high- and low-risk GC patients. **(D)** ROC curves for the nomogram in the prediction of 5-, 6-, and 7-year OS probabilities. **(E)** Decision curve analysis curves of the nomogram for OS outcomes.

A prognostic nomogram was established by integrating the TNFα-derived gene signature, age, T stage, N stage, M stage, and pathological stage, which might be predictive of BC patients’ survival outcomes through a quantitative scoring method (Figure 4A). In accordance with the nomogram, each patient would obtain a total point from each prognostic indicator. Calibration curves demonstrated that the predictive accuracy of this nomogram was similar to the actual OS outcomes (Figure 4B). With the median value of the nomogram score, GC patients were clustered into high- and low-risk groups. It was found that high-risk patients were indicative of more undesirable OS outcomes than low-risk patients (Figure 4C). ROCs at 5-, 6-, and 7-year OS demonstrated that this nomogram displayed excellent efficacy in the prediction of OS outcomes (Figure 4D). Additionally, decision curve analyses demonstrated that the nomogram had a remarkable advantage of the TNFα-derived gene signature alone and displayed a high potential for clinical utility (Figure 4E).

### Signal Pathways Involved in the Tumor Necrosis Factor-α–Derived Gene Signature

**FIGURE 5:**
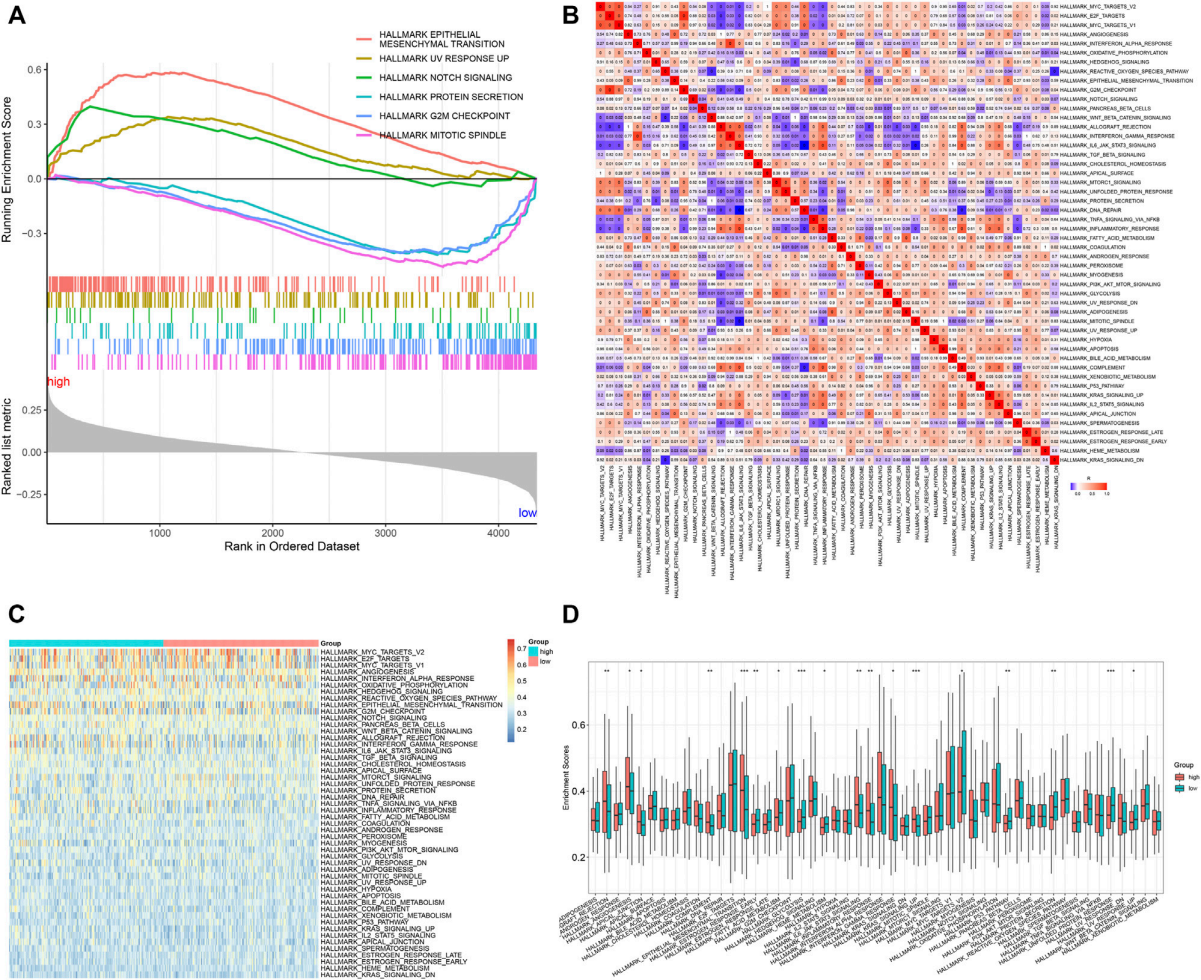
Signal pathways involved in the TNFα-derived gene signature. **(A)** GSEA for the differential hallmark gene sets between high- and low-risk GC specimens. **(B)** Heatmap showing the interactions between hallmark gene sets across GC specimens. **(C)** Heatmap showing the activities of hallmark gene sets in two groups. **(D)** Comparison of the activities of hallmark gene sets between groups. *p < 0.05; **p < 0.01; ***p < 0.001.

It was found that the epithelial-mesenchymal transition, UV response up, and Notch signaling presented enhanced activities in high-risk specimens, in accordance with GSEA results (Figure 5A). Meanwhile, protein secretion, G2M checkpoint, and mitotic spindle exhibited reduced activities in low-risk specimens. Moreover, we quantified the activities of ssGSEA gene sets in each GC specimen (Figure 5B). Compared with the low-risk group, graft rejection, angiogenesis, apical junction, complement, epithelial-mesenchymal transition, IL6-JAK-STAT3 signaling, inflammatory response, interferon-gamma response, and KRAS signaling showed remarkedly enhanced activities in the high-risk group (Figures 5C,D). Oppositely, late estrogen response, glycolysis, heme metabolism, MYC targets V2, p53 pathway, protein secretion, unfolded protein response, and UV response up had prominently reduced activities in the high-risk group.

### Heterogeneity in Drug Responses and Somatic Mutations Between High- and Low-Risk Groups

**FIGURE 6:**
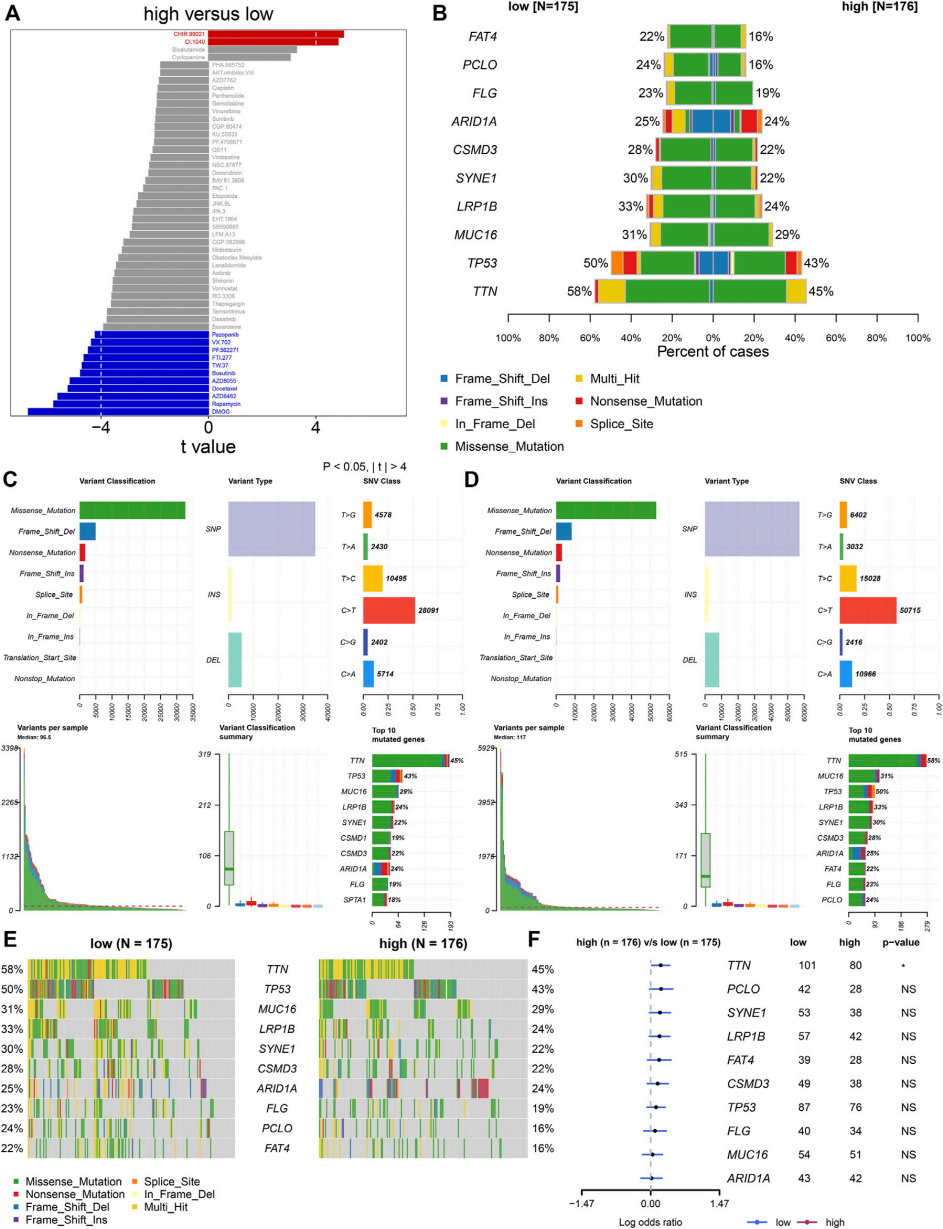
TNFα-derived genomic model-relevant drug responses and somatic mutations. **(A)** Comparing drug responses between high- and low-risk groups. **(B)** Distribution of the first ten mutated genes in high- and low-risk patients. **(C,D)** Landscape of somatic mutations in high- and low-risk patients. **(E)** Oncoplots for the first ten frequently mutated genes in two groups. **(F)** Forest plots showing the differences in mutated genes between groups.

Furthermore, analyses were presented for investigation of the difference in responses to small molecular agents between groups. Our study noted that CHIR.99021 and CI.1040 displayed higher IC50 values in high- than low-risk patients (Figure 6A). Additionally, high-risk patients showed reduced IC50 values of pazopanib, VX.702, PF.562271, FTI.277, TW.37, bosutinib, AZD8055, docetaxel, AZD6482, rapamycin, and DMOG in comparison to low-risk patients. The aforementioned data suggested that low-risk patients presented higher sensitivity to CHIR.99021 and CI.1040, while high-risk patients displayed enhanced responses to pazopanib, VX.702, PF.562271, FTI.277, TW.37, bosutinib, AZD8055, docetaxel, AZD6482, rapamycin, and DMOG. We also compared the differences in somatic mutations between high- and low-risk groups. The first ten mutated genes included TTN, TP53, MUC16, LRP1B, SYNE1, CSMD3, ARID1A, FLG, PCLO, and FAT4. Higher mutational frequencies of the aforementioned genes were observed in low- than high-risk groups (Figure 6B). Both in high- and low-risk groups, missense mutation was the most frequent mutational type (Figures 6C–E). In particular, there was a significant difference in TTN mutation between groups (Figure 6F).

### Heterogeneity in Immune Cell Infiltrations Between High- and Low-Risk Groups

**FIGURE 7:**
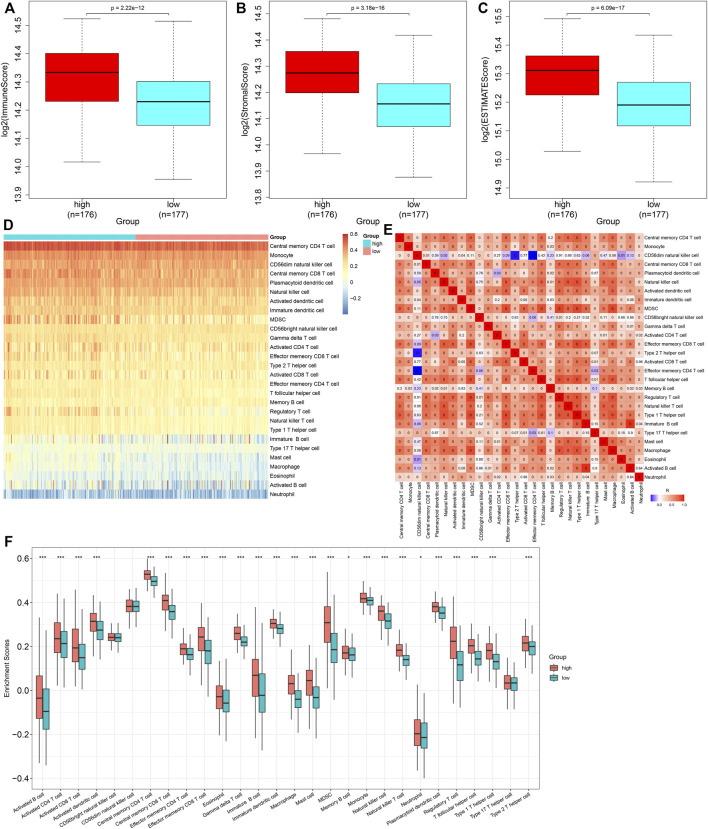
Heterogeneity in immune cell infiltrations between high- and low-risk subgroups. **(A–C)** Comparison of immune and stromal scores, as well as ESTIMATE scores, between groups. **(D)** Distribution of abundance levels of immune cells across GC tissues. **(E)** Heatmap visualizing the interactions of diverse immune cells across GC tissues. **(F)** Comparison of abundance levels of immune subpopulations between groups. *p < 0.05; ***p < 0.001.

Through the ESTIMATE algorithm, we estimated the infiltration levels of immune and stromal cells. As a result, the high-risk score was in relation to increased immune and stromal scores, as well as the ESTIMATE score (Figures 7A–C). The abundance levels of immune cells were quantified within GC tissues by the ssGSEA method. There were enhanced abundance levels of activated B cells, activated CD4 T cells, activated CD8 T cells, activated dendritic cells, central memory CD4 T cells, central memory CD8 T cells, effector memory CD4 T cells, effector memory CD8 T cells, eosinophils, gamma delta T cells, immature B cells, immature dendritic cells, macrophages, mast cells, MDSCs, memory B cells, natural killer T cells, neutrophils, plasmacytoid dendritic cells, regulatory T cells, T follicular helper cells, type 1 helper cells, and type 2 helper cells in high- compared with low-risk specimens (Figures 7D–F).

### Association of the Tumor Necrosis Factor-α–Derived Gene Signature With Immune Response

**FIGURE 8:**
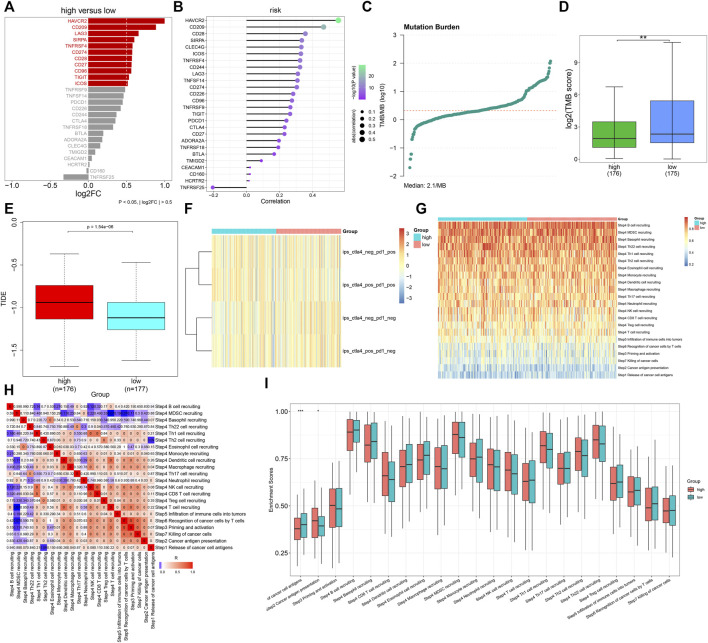
Association of the TNFα-derived gene signature with immune response in GC. **(A)** Comparing the expressions of immune checkpoint molecules between groups. **(B)** Associations of immune checkpoints with the TNFα-derived risk score across GC specimens. **(C)** Distribution of TMB scores among GC tissues. **(D)** Comparison of the TMB score between subgroups. **p < 0.01. **(E)** Comparison of the TIDE score between two groups. **(F)** Distribution of IPS scores in two subgroups. **(G)** Heatmap depicting the activities of the cancer immunity cycle across GC specimens. **(H)** Associations of all steps within the cancer immunity cycle across GC specimens. **(I)** Differences in the activities of the cancer immunity cycle between high- and low-risk groups. *p < 0.05; ***p < 0.001.

Further analyses uncovered that immune checkpoint molecules containing HAVCR2, CD209, LAG3, SIRPA, TNFRSF4, CD274, CD28, CD27, CD96, TIGIT, and ICOS displayed enhanced expressions in high- compared with low-risk groups (Figure 8A). Additionally, the TNFα-derived risk score was positively associated with most immune checkpoint molecules (Figure 8B). We calculated the TMB score across GC tissues, with a median value of 2.1/MB (Figure 8C). A higher TMB score was investigated in low- than high-risk patients (Figure 8D). Moreover, we noted that high-risk patients presented elevated TIDE scores (Figure 8E). Nevertheless, no prominent difference in the IPS score was noted between groups (Figure 8F). The activities of all steps within the cancer immunity cycle were estimated in GC tissues (Figures 8G,H). In particular, there were reduced activities of the release of cancer cell antigens and enhanced activities of cancer antigen presentation in high- than low-risk groups (Figure 8I). The aforementioned data were indicative that the TNFα-derived genomic model might be applied as a predictor of immune responses in GC.

### Experimental Verification of the Tumor Necrosis Factor-α-Derived Gene Signature

**FIGURE 9:**
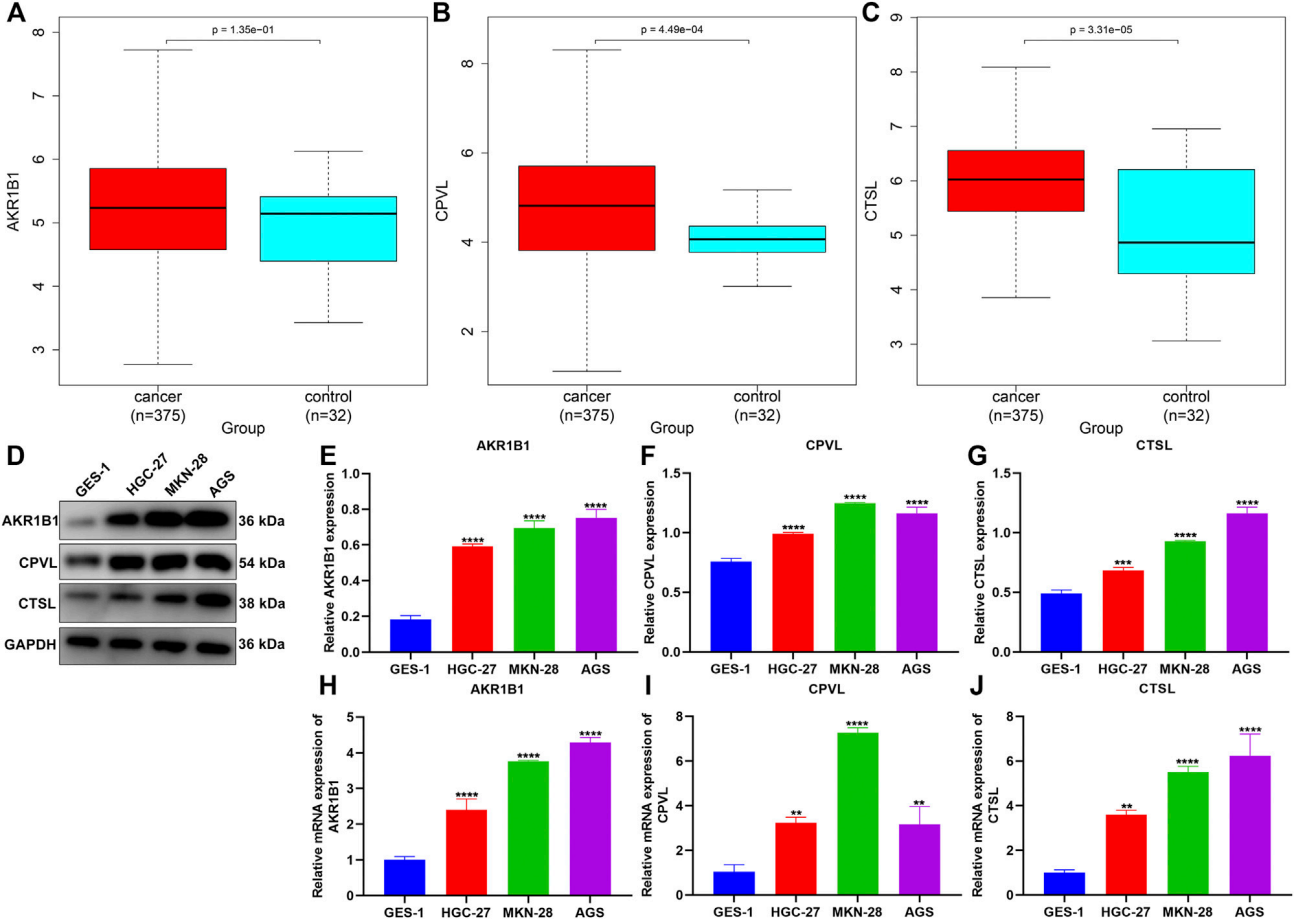
Experimental verification of the expression of genes in the TNFα-derived gene signature. **(A–C)** Comparison of the expressions of AKR1B1, CPVL, and CTSL between GC and normal tissues. **(D–G)** Western blotting for validation of the expressions of AKR1B1, CPVL, and CTSL in human gastric mucosal cells GES-1 and human GC cells HGC-27, MKN-28, and AGS. **(H–J)** RT-qPCR for verification of the expressions of AKR1B1, CPVL, and CTSL in human gastric mucosal cells GES-1 and human GC cells HGC-27, MKN-28, and AGS. **p < 0.01; ***p < 0.001; ****p < 0.0001.

We noted that AKR1B1, CPVL, and CTSL within the TNFα-derived gene signature presented remarkably increased expressions in GC than in normal tissues (Figures 9A–C). Their expressions were further verified in human gastric mucosal cells GES-1 and human GC cells HGC-27, MKN-28, and AGS. Our data confirmed the significant upregulation of AKR1B1, CPVL, and CTSL in HGC-27, MKN-28, and AGS cells than GES-1 cells (Figures 9D–J).

## Discussion

Through ssGSEA, we quantified the activities of hallmark gene sets in GC. Among them, TNFα signaling acted as a prognostic indicator of GC. Thereafter, TNFα-derived genes were identified with the WGCNA algorithm. With the LASSO algorithm, a TNFα-derived gene signature composed of AKR1B1, CPVL, and CTSL was developed for GC. Survival analyses uncovered that this signature might enable the estimation of patients’ OS, DSS, DFS, and PFS outcomes. Time-independent ROC curves and multivariate Cox regression models confirmed the reliability and independence of the TNFα-derived gene signature in predicting GC outcomes. Additionally, this signature was in relation to more severe histological grades and pathological stages of GC patients, indicating that it contributed to GC progression. Meta-analyses have demonstrated the associations of TNFα alterations with GC risks (Wang et al., 2016).

We noted the prominent activities of stromal activation-relevant signaling like epithelial-mesenchymal transition (EMT) (Zhu et al., 2019), angiogenesis, immune activation-relevant pathways such as graft rejection, complement, IL6-JAK-STAT3 signaling, and inflammatory response, as well as carcinogenic pathways such as Notch signaling and KRAS signaling in high-risk GC patients. Experimental evidence suggests that TNF-α triggers invasion and metastases of GC through downregulation of pentraxin 3 (Cui et al., 2020). TNF-α induces EMT in GC cells *via* activating IL-6/STAT3 signaling (Chen et al., 2017). More frequent somatic mutations were investigated in low-risk patients, and enhanced immune cell infiltrations and immune checkpoint expressions were detected in high-risk patients. Enhanced mast cells trigger immunosuppression in GC *via* TNF-α-PD-L1 signaling (Lv et al., 2019). Tumor-associated macrophages facilitate the PD-L1 expression in GC *via* IL-6 and TNF-α signals (Ju et al., 2020). Cancer pain is one of the clinical symptoms with a high incidence in cancer patients. As estimated, patients with moderate and severe cancer pain account for 75%∼90% (Scarborough and Smith, 2018). Animal pain experiments have confirmed that TNF-α is positively correlated with animal pain performance (Yang et al., 2020). Evidence has also shown that TNF-α is the key link that causes cancer pain in cancer patients (Ling et al., 2020). TNF-α can activate NF-kB, NGF, and other signaling pathways, and at the same time, it also plays a positive feedback effect on its production (Yoneda et al., 2021). Moreover, the activated signaling pathways can cause the sensitization of downstream nerve cells and cause pain. Therefore, the application of bioinformatics to help achieve accurate prediction, prevention, and reduction of the symptoms of cancer pain in patients with gastric cancer might be an effective approach for future enhancing scientists to explore the precision nursing of cancer symptoms.

Our experimental results confirmed the significant upregulation of AKR1B1, CPVL, and CTSL in human GC cells HGC-27, MKN-28, and AGS compared with human gastric mucosal cells GES-1. Previously, AKR1B1 expression has been remarkably upregulated in GC than in nontumor tissues (Li et al., 2020). Additionally, it displays remarkable associations with survival outcomes and immune cell infiltration in GC (Zhou et al., 2021). CPVL upregulation has been proposed in GC over non-cancerous specimens (Ran et al., 2015). CTSL triggers angiogenesis through modulating the CDP/Cux/VEGF-D pathway in GC (Pan et al., 2020). These findings suggested the critical functions of AKR1B1, CPVL, and CTSL in gastric carcinogenesis.

A few limitations should be pointed out in our study. All data utilized in our study were curated from public cohorts. Although GC patients were randomly assigned to training and testing sets, the internal verification method was of only limited value. In-depth external verification was of importance for confirming and expanding our discovery as an approach for the development of a clinically worthy prognostic model. Moreover, our evaluation of the associations of the TNFα-derived gene signature with GC patients’ clinicopathological features was not exhaustive. In accordance with the limitations, currently, the TNFα-derived gene signature we established is of only limited clinical utility and required extensive verification.

## Conclusion

Collectively, our research uncovered the implication of the TNFα-derived gene signature in predicting prognosis, immune escape, and genomic mutations in GC, which might display regimens for enhancing the immunotherapeutic responses. This signature as a reliable prognostic and immunotherapeutic predictor might guide clinical nursing management and personalized medicine.

## Data Availability

The original contributions presented in the study are included in the article/Supplementary Material; further inquiries can be directed to the corresponding author.

## Supplementary Material

Supplementary Table S1 List of the genes in the “tan” module.

## Abbreviations

DFS, disease-free survival; DSS, disease-specific survival; DCA, decision curve analyses; ESTIMATE, Estimation of STromal and Immune cells in MAlignant Tumor tissues using Expression data; GSVA, gene set variation analysis; GC, gastric cancer; IPS, immunophenoscore; IC50, half-maximal inhibitory concentration; LASSO, least absolute shrinkage and selection operator; OS, overall survival; PFS, progression-free survival; ROC, receiver operating characteristic; TIDE, T-cell dysfunction and exclusion; TMB, tumor mutational burden; qRT-PCR, reverse transcription and quantitative real-time PCR; TNF-α, tumor necrosis factor-alpha; ssGSEA, single-sample gene set enrichment analysis; WGCNA, weighted gene co-expression network analysis.

## References

[B1] Baj J. Korona-Głowniak I. Forma A. Maani A. Sitarz E. Rahnama-Hezavah M. (2020). Mechanisms of the Epithelial-Mesenchymal Transition and Tumor Microenvironment in Helicobacter Pylori-Induced Gastric Cancer. Cells 9 (4), 1055. 10.3390/cells9041055 PMC7225971 32340207

[B2] Bray F. Ferlay J. Soerjomataram I. Siegel R. L. Torre L. A. Jemal A. (2018). Global Cancer Statistics 2018: GLOBOCAN Estimates of Incidence and Mortality Worldwide for 36 Cancers in 185 Countries. CA Cancer J. Clin. 68 (6), 394–424. 10.3322/caac.21492 30207593

[B3] Chalmers Z. R. Connelly C. F. Fabrizio D. Gay L. Ali S. M. Ennis R. (2017). Analysis of 100,000 Human Cancer Genomes Reveals the Landscape of Tumor Mutational Burden. Genome Med. 9 (1), 34. 10.1186/s13073-017-0424-2 28420421 PMC5395719

[B4] Charoentong P. Finotello F. Angelova M. Mayer C. Efremova M. Rieder D. (2017). Pan-cancer Immunogenomic Analyses Reveal Genotype-Immunophenotype Relationships and Predictors of Response to Checkpoint Blockade. Cell Rep. 18 (1), 248–262. 10.1016/j.celrep.2016.12.019 28052254

[B5] Chen D. S. Mellman I. (2013). Oncology Meets Immunology: The Cancer-Immunity Cycle. Immunity 39 (1), 1–10. 10.1016/j.immuni.2013.07.012 23890059

[B6] Chen G. Tang N. Wang C. Xiao L. Yu M. Zhao L. (2017). TNF-α-inducing Protein of *Helicobacter pylori* Induces Epithelial-Mesenchymal Transition (EMT) in Gastric Cancer Cells through Activation of IL-6/STAT3 Signaling Pathway. Biochem. Biophys. Res. Commun. 484 (2), 311–317. 10.1016/j.bbrc.2017.01.110 28130110

[B7] Chen L. Wang G. Qiao X. Wang X. Liu J. Niu X. (2020). Downregulated miR-524-5p Participates in the Tumor Microenvironment of Ameloblastoma by Targeting the Interleukin-33 (IL-33)/Suppression of Tumorigenicity 2 (ST2) Axis. Med. Sci. Monit. 26, e921863. 10.12659/msm.921863 31990904 PMC6998793

[B8] Cui X. Zhang H. Cao A. n. Cao L. Hu X. (2020). Cytokine TNF-α Promotes Invasion and Metastasis of Gastric Cancer by Down-Regulating Pentraxin3. J. Cancer 11 (7), 1800–1807. 10.7150/jca.39562 32194791 PMC7052870

[B9] Engebretsen S. Bohlin J. (2019). Statistical Predictions with Glmnet. Clin. Epigenet. 11 (1), 123. 10.1186/s13148-019-0730-1 PMC6708235 31443682

[B10] Geeleher P. Cox N. Huang R. S. (2014a). pRRophetic: An R Package for Prediction of Clinical Chemotherapeutic Response from Tumor Gene Expression Levels. PLoS One 9 (9), e107468. 10.1371/journal.pone.0107468 25229481 PMC4167990

[B11] Geeleher P. Cox N. J. Huang R. (2014b). Clinical Drug Response can be Predicted Using Baseline Gene Expression Levels and *In Vitro* Drug Sensitivity in Cell Lines. Genome Biol. 15 (3), R47. 10.1186/gb-2014-15-3-r47 24580837 PMC4054092

[B12] Hänzelmann S. Castelo R. Guinney J. (2013). GSVA: Gene Set Variation Analysis for Microarray and RNA-Seq Data. BMC Bioinforma. 14, 7. 10.1186/1471-2105-14-7 PMC3618321 23323831

[B13] Ishimoto T. Miyake K. Nandi T. Yashiro M. Onishi N. Huang K. K. (2017). Activation of Transforming Growth Factor Beta 1 Signaling in Gastric Cancer-Associated Fibroblasts Increases Their Motility, via Expression of Rhomboid 5 Homolog 2, and Ability to Induce Invasiveness of Gastric Cancer Cells. Gastroenterology 153 (1), 191–204. 10.1053/j.gastro.2017.03.046 28390866

[B14] Jiang P. Gu S. Pan D. Fu J. Sahu A. Hu X. (2018). Signatures of T Cell Dysfunction and Exclusion Predict Cancer Immunotherapy Response. Nat. Med. 24 (10), 1550–1558. 10.1038/s41591-018-0136-1 30127393 PMC6487502

[B15] Ju X. Zhang H. Zhou Z. Chen M. Wang Q. (2020). Tumor-associated Macrophages Induce PD-L1 Expression in Gastric Cancer Cells through IL-6 and TNF-ɑ Signaling. Exp. Cell Res. 396 (2), 112315. 10.1016/j.yexcr.2020.112315 33031808

[B16] Kim W. Chu T. H. Nienhüser H. Jiang Z. Del Portillo A. Remotti H. E. (2021). PD-1 Signaling Promotes Tumor-Infiltrating Myeloid-Derived Suppressor Cells and Gastric Tumorigenesis in Mice. Gastroenterology 160 (3), 781–796. 10.1053/j.gastro.2020.10.036 33129844 PMC7878361

[B17] Langfelder P. Horvath S. (2008). WGCNA: An R Package for Weighted Correlation Network Analysis. BMC Bioinform. 9, 559. 10.1186/1471-2105-9-559 PMC2631488 19114008

[B18] Li X. Yang J. Gu X. Xu J. Li H. Qian J. (2020). The Expression and Clinical Significance of Aldo-Keto Reductase 1 Member B1 in Gastric Carcinoma. DNA Cell Biol. 39 (7), 1322–1327. 10.1089/dna.2020.5550 32412859

[B19] Liberzon A. Birger C. Thorvaldsdóttir H. Ghandi M. Mesirov J. P. Tamayo P. (2015). The Molecular Signatures Database Hallmark Gene Set Collection. Cell Syst. 1 (6), 417–425. 10.1016/j.cels.2015.12.004 26771021 PMC4707969

[B20] Ling D. Zhao Y. Zhang Z. Li J. Zhu C. Wang Z. (2020). Morphine Inhibits the Promotion of Inflammatory Microenvironment on Chronic Tibial Cancer Pain through the PI3K-Akt-NF-κB Pathway. Am. J. Transl. Res. 12 (10), 6868–6878. 33194078 PMC7653610

[B21] Lv Y. Zhao Y. Wang X. Chen N. Mao F. Teng Y. (2019). Increased Intratumoral Mast Cells Foster Immune Suppression and Gastric Cancer Progression through TNF-α-PD-L1 Pathway. J. Immunother. Cancer 7 (1), 54. 10.1186/s40425-019-0530-3 30808413 PMC6390584

[B22] Mayakonda A. Lin D.-C. Assenov Y. Plass C. Koeffler H. P. (2018). Maftools: Efficient and Comprehensive Analysis of Somatic Variants in Cancer. Genome Res. 28 (11), 1747–1756. 10.1101/gr.239244.118 30341162 PMC6211645

[B23] Pan T. Jin Z. Yu Z. Wu X. Chang X. Fan Z. (2020). Cathepsin L Promotes Angiogenesis by Regulating the CDP/Cux/VEGF-D Pathway in Human Gastric Cancer. Gastric Cancer 23 (6), 974–987. 10.1007/s10120-020-01080-6 32388635 PMC7567730

[B24] Qiu X.-T. Song Y.-C. Liu J. Wang Z.-M. Niu X. He J. (2020). Identification of an Immune-Related Gene-Based Signature to Predict Prognosis of Patients with Gastric Cancer. World J. Gastrointest. Oncol. 12 (8), 857–876. 10.4251/wjgo.v12.i8.857 32879664 PMC7443845

[B25] Ran X. Xu X. Yang Y. She S. Yang M. Li S. (2015). A Quantitative Proteomics Study on Olfactomedin 4 in the Development of Gastric Cancer. Int. J. Oncol. 47 (5), 1932–1944. 10.3892/ijo.2015.3168 26398045

[B26] Saeed A. Park R. Sun W. (2021). The Integration of Immune Checkpoint Inhibitors with VEGF Targeted Agents in Advanced Gastric and Gastroesophageal Adenocarcinoma: A Review on the Rationale and Results of Early Phase Trials. J. Hematol. Oncol. 14 (1), 13. 10.1186/s13045-021-01034-0 33436042 PMC7802258

[B27] Scarborough B. M. Smith C. B. (2018). Optimal Pain Management for Patients with Cancer in the Modern Era. CA Cancer J. Clin. 68 (3), 182–196. 10.3322/caac.21453 29603142 PMC5980731

[B28] Subramanian A. Tamayo P. Mootha V. K. Mukherjee S. Ebert B. L. Gillette M. A. (2005). Gene Set Enrichment Analysis: A Knowledge-Based Approach for Interpreting Genome-wide Expression Profiles. Proc. Natl. Acad. Sci. U.S.A. 102 (43), 15545–15550. 10.1073/pnas.0506580102 16199517 PMC1239896

[B29] Wang P. Wang J. Yu M. Li Z. (2016). Tumor Necrosis Factor-α T-857C (Rs1799724) Polymorphism and Risk of Cancers: A Meta-Analysis. Dis. Markers 2016, 1–9. 10.1155/2016/4580323 PMC5223007 28115787

[B30] Wang X. Cheng G. Miao Y. Qiu F. Bai L. Gao Z. (2021). Piezo Type Mechanosensitive Ion Channel Component 1 Facilitates Gastric Cancer Omentum Metastasis. J. Cell Mol. Med. 25 (4), 2238–2253. 10.1111/jcmm.16217 33439514 PMC7882944

[B31] Wu L. Zhao N. Zhou Z. Chen J. Han S. Zhang X. (2021). PLAGL2 Promotes the Proliferation and Migration of Gastric Cancer Cells via USP37-Mediated Deubiquitination of Snail1. Theranostics 11 (2), 700–714. 10.7150/thno.47800 33391500 PMC7738862

[B32] Yang B. Zhang Z. Yang Z. Ruan J. Luo L. Long F. (2020). Chanling Gao Attenuates Bone Cancer Pain in Rats by the IKKβ/NF-κB Signaling Pathway. Front. Pharmacol. 11, 525. 10.3389/fphar.2020.00525 32431607 PMC7214814

[B33] Yoneda T. Hiasa M. Okui T. Hata K. (2021). Sensory Nerves: A Driver of the Vicious Cycle in Bone Metastasis? J. Bone Oncol. 30, 100387. 10.1016/j.jbo.2021.100387 34504741 PMC8411232

[B34] Yoshihara K. Shahmoradgoli M. Martínez E. Vegesna R. Kim H. Torres-Garcia W. (2013). Inferring Tumour Purity and Stromal and Immune Cell Admixture from Expression Data. Nat. Commun. 4, 2612. 10.1038/ncomms3612 24113773 PMC3826632

[B35] Yu L. Cao C. Li X. Zhang M. Gu Q. Gao H. (2021). Complete Loss of miR-200 Family Induces EMT Associated Cellular Senescence in Gastric Cancer. Oncogene 41, 26–36. 10.1038/s41388-021-02067-y 34667277 PMC8724006

[B36] Zhang M. Hu S. Min M. Ni Y. Lu Z. Sun X. (2021). Dissecting Transcriptional Heterogeneity in Primary Gastric Adenocarcinoma by Single Cell RNA Sequencing. Gut 70 (3), 464–475. 10.1136/gutjnl-2019-320368 32532891 PMC7873416

[B37] Zhou Q. Wu X. Wang X. Yu Z. Pan T. Li Z. (2020). The Reciprocal Interaction between Tumor Cells and Activated Fibroblasts Mediated by TNF-α/IL-33/ST2L Signaling Promotes Gastric Cancer Metastasis. Oncogene 39 (7), 1414–1428. 10.1038/s41388-019-1078-x 31659258 PMC7018661

[B38] Zhou W. Wu C. Zhao C. Huang Z. Lu S. Fan X. (2021). An Advanced Systems Pharmacology Strategy Reveals AKR1B1, MMP2, PTGER3 as Key Genes in the Competing Endogenous RNA Network of Compound Kushen Injection Treating Gastric Carcinoma by Integrated Bioinformatics and Experimental Verification. Front. Cell Dev. Biol. 9, 742421. 10.3389/fcell.2021.742421 34646828 PMC8502965

[B39] Zhu X. Chen L. Liu L. Niu X. (2019). EMT-Mediated Acquired EGFR-TKI Resistance in NSCLC: Mechanisms and Strategies. Front. Oncol. 9, 1044. 10.3389/fonc.2019.01044 31681582 PMC6798878

[B40] Zhuang H. Dai X. Zhang X. Mao Z. Huang H. (2020). Sophoridine Suppresses Macrophage-Mediated Immunosuppression through TLR4/IRF3 Pathway and Subsequently Upregulates CD8^+^ T Cytotoxic Function against Gastric Cancer. Biomed. Pharmacother. 121, 109636. 10.1016/j.biopha.2019.109636 31733580

